# The characteristics of stroke units in Ontario: a pan-provincial survey

**DOI:** 10.1186/s12913-017-2099-1

**Published:** 2017-02-21

**Authors:** Valeria E. Rac, Yeva Sahakyan, Iris Fan, Luciano Ieraci, Ruth Hall, Linda Kelloway, Gabrielle van der Velde, Moira K. Kapral, Mark Bayley, Murray Krahn

**Affiliations:** 1 Toronto Health Economics and Technology Assessment (THETA) Collaborative, Toronto General Research Institute, Toronto General Hospital, University Health Network, University of Toronto, Toronto, ON Canada; 2 Institute of Health Policy, Management and Evaluation (IHPME), University of Toronto, Toronto, ON Canada; 3 Cancer Care Ontario, Toronto, ON Canada; 4 Institute for Clinical Evaluative Sciences (ICES), Toronto, ON Canada; 5 Ontario Stroke Network, Toronto, ON Canada; 6 Leslie Dan Faculty of Pharmacy, University of Toronto, Toronto, ON Canada; 7 Toronto General Hospital, University Health Network, University of Toronto, Toronto, ON Canada; 8 Faculty of Medicine, University of Toronto, Toronto, ON Canada; 9 Toronto Rehab Foundations, University Health Network, Toronto, ON Canada

**Keywords:** Stoke units, Core component, Environmental scan, Ontario

## Abstract

**Background:**

Previous studies have demonstrated that organized, multidisciplinary care is the cornerstone of current strategies to reduce the death and disability caused by stroke. Identification of stroke units and an understanding of their composition and operation would provide insight for the further actions required to improve stroke care. The objective of this study was to identify and survey stroke units in Canada’s largest province, Ontario (population of 13 million) in order to describe availability, structure, staffing, processes of care, and type of population stroke units serve.

**Methods:**

The Ontario Stroke Network (2011) list of stroke units and snowball sampling was used to identify all stroke units. During 2013 – 2014 an interviewer conducted telephone surveys with the stroke unit managers using closed and semi-open ended questions. Descriptive statistics were used to summarize survey responses.

**Results:**

The survey identified 32 stroke units, and a respondent from every stroke unit (100% response rate) was interviewed. Twenty one were acute stroke units, 10 were integrated stroke units and one was classified as a rehabilitation stroke unit. Stroke units were available in all 14 Local Health Integration Networks except Central West. The estimated average number of stroke patients served per stroke unit was 604 with six-fold variation (242 to 1480) across the province. The typical population served in stroke units were patients with either ischemic or hemorrhagic stroke. Data consistently reported on the processes of stroke care, including the availability of multidisciplinary staff, specific diagnostic imaging, use of validated assessment tools, and the delivery of patient education. Details about the core components of stoke care were provided by 16 stroke units (50%).

**Conclusions:**

This study demonstrates the heterogeneous structure of stroke units in Ontario and signaled potential disparity in access to stroke units. Many core components are in place, but half of the stroke units in Ontario do not meet all criteria. Areas for potential improvement include stroke care training for the multidisciplinary team, provision of individualized rehabilitation plans, and early discharge assessment.

**Electronic supplementary material:**

The online version of this article (doi:10.1186/s12913-017-2099-1) contains supplementary material, which is available to authorized users.

## Background

Stroke is a major health challenge, placing a substantial burden on patients, families and health care systems. Each year 15 million people suffer a stroke worldwide, where nearly six million die and another five million experience long-term disability [1]. In Canada, stroke is the third leading cause of death [1]. It imposes a significant cost to the Canadian economy with $3.6 billion spent annually in hospital and physician services, lost wages, and decreased productivity [1]. Due to the aging population, the absolute number of individuals at risk is projected to increase [2]. According to the World Health Organization, disability-adjusted life years lost to stroke will increase from 38 to 61 million between 1990 and 2020 [3].

Organized care is the cornerstone of current strategies to reduce mortality and disability caused by stroke. Randomized controlled clinical trials have shown a reduction in mortality, dependency and institutionalized care for patients treated in stroke units (SUs) relative to patients who received care in general wards [4, 5]. The effect observed in clinical trials was replicated in routine practice as well. Seenan et al. [6] carried out a systematic review of 25 observational studies comparing death and other complications of stroke patients managed in SUs versus non-SU care. Results showed that patients experienced better survival (mortality odds ratio (OR) 0.79, 95% confidence interval (CI) 0.73–0.86) within 1 year of a stroke episode if they were treated in a SU.

In 2006, Canadian Best Practice Recommendations providing evidence-based practices for stroke management were released [7]. According to that document, a SU should consist of a geographically defined unit, dedicated multidisciplinary team with specialized expertise in stroke and have standardized processes of care and resources [7, 8]. This was followed by “A Guide to the Implementation of Stroke Unit Care”, which focused on a set of considerations for the development and enhancement of SUs [9]. Nonetheless, implementation of SUs in Canada in comparison with other countries is low. According to the Canadian Stroke Network report (2011), only 23% of stroke patients were treated in SUs [10], compared with >80% in Sweden (2006) [11], 75% in England (2008), and 50% in Australia (2009) [12].

Considering that implementation of SUs is a dynamic process, and that not all components of effective SUs are applicable or feasible for all facilities [9], it is important to identify the availability of SUs and the degree in which core components are currently in place. Determining the characteristics of SUs in Ontario, as well as their effectiveness and cost-effectiveness in a pragmatic setting, was the impetus for the project that was funded by the Ontario Stroke Network (OSN) and the Ontario Ministry of Health and Long-Term Care. The specific research objective of this study was to conduct a pan-provincial survey of existing SUs in the province of Ontario to describe their availability, structure, the care they provide, and the population they serve.

## Methods

The Ontario Stroke Unit survey was designed to collect data on characteristics of SUs. In order to identify all SUs in Ontario, we used the OSN database (2011) [13], followed by a snowball sampling technique. During snowball sampling, all respondents were asked whether they were aware of the existence of any other Ontario SUs [14].

A closed- and semi open-ended questionnaire, based on Canadian Best Practice Recommendations for Stroke Care [8] and Langhorne’s findings on the effective components of SU care [15] (Additional file 1), was designed. Following an iterative process, the questionnaire was piloted, and once finalized, it was administered to the clinical and administrative leaders (managers, medical directors and team leaders) of the identified SUs. The questionnaire focused on: i) characteristics of SUs, such as structure and organization (geographic location, size and function of the unit); ii) staffing components (multidisciplinary team composition, their expertise, staffing levels and intensity); and iii) processes of care (patient population, diagnostic services, implementation of standardized protocols and valid assessment tools, rehabilitation and patient education).

An interviewer conducted a telephone survey with the SU managers in 2013-2014. The average survey duration was approximately 60 min per SU.

### Definition and regional distribution of SUs

In this study we used the Canadian Stroke Strategy definition of SUs [9]:

I. Acute Stroke Unit (ASU): A specialized, geographically defined hospital unit dedicated to the management of stroke patients during the first seven to 10 days, or longer, following an acute stroke event and staffed by an interprofessional team.
II. Integrated Stroke Unit (ISU): A specialized, geographically defined hospital unit dedicated to the management of stroke patients. The unit provides both acute and rehabilitation care to patients during their inpatient stays following a stroke.
III. Rehabilitation Stroke Unit (RSU): A specialized, geographically defined rehabilitation unit dedicated to the rehabilitation of stroke patients.

Dedicated SU staff members were defined as someone who wase exclusively assigned to a SU. Dedicated beds were defined as beds that were exclusively allocated to stroke patients.

In Ontario there are 14 Local Health Integrational Networks (LHINs), which are responsible for centralized administration of health services within the province. We used boundaries of each LHIN to estimate geographic distribution of SUs [16]. The number of available SUs and stroke incident cases per LHIN (2012-2013) were used to approximate potential volume of stroke patients treated per SU [17].

### Statistical analyses

Statistical analyses were conducted using SAS version 9.4 (SAS Institute Inc, Cary, North Carolina). Descriptive statistics were used to summarize survey responses. Continuous data were described using means/medians and standard deviations/interquartile range. Categorical data were summarized as frequency counts and percentages.

## Results

Overall, 32 SUs were identified (21 ASUs, 10 ISUs and one RSU) (Additional file 2). Two of these were identified using the snowball sampling method. At least one respondent from each SU was interviewed (100% response rate), with a total of 39 staff members interviewed across 32 SUs.

**Fig. 1:**
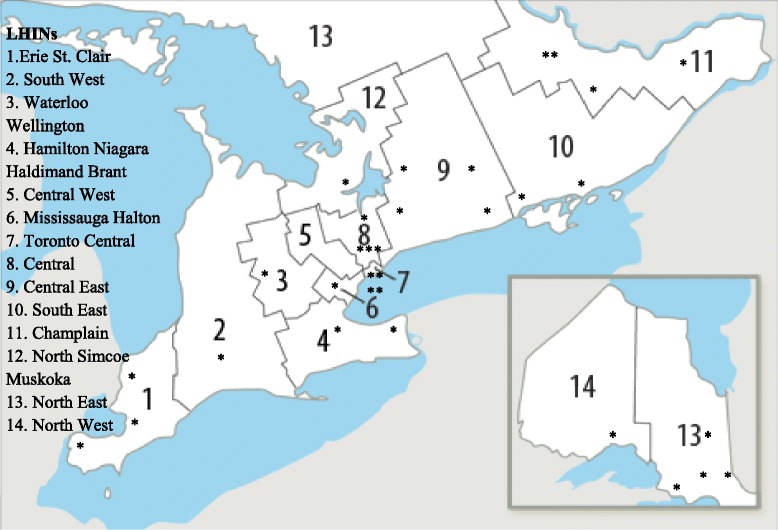
Stroke units’ distribution across LHINs in Ontario The map has been modified from http://www.lhins.on.caCopyright information: ©Queen's Printer for Ontario, 2014. Written permission was granted by the copyright holder

Surveys were conducted with program/unit managers (*n* = 13), clinic managers (*n* = 11), patient care managers (*n* = 4), or other administrative staff (*n* = 4). The additional staff members (*n* = 7) were aided in answering the questionnaire if required. SUs were available in all 14 LHINs except Central West (Fig. 1). The estimated average ratio of stroke patients served per SU was 604, with wide variability across the province [242 in the North East LHIN to 1480 in the Mississauga Halton LHIN (Table 1)]. An overwhelming majority (*n* = 24; 75%) of SUs were in operation for more than 5 years with an average duration of operation of 7.3 ± 4.4 years.

**Table 1:** Geographic distribution of stroke units and estimated access to care

LHIN	# of SU	Total population	# of stroke/TIA patients (2012-13)^a^	# of stroke/TIA patients per SU
Erie St. Clair	3	623,300	1,219	407
South West	1	890,100	1,376	1,376
HNHB	2	1,298,300	2,544	1,272
Waterloo Wellington	1	679,700	996	996
Mississauga Halton	1	1,002,300	1,480	1,480
Central West	0	735,200	805	N/A
Central	4	1,522,800	1,734	434
Central East	4	1,419,800	2,052	513
Toronto Central	4	1,075,100	2,464	616
North Simcoe Muskoka	1	417,000	858	858
South East	2	457,200	861	431
Champlain	4	1,131,000	1,821	456
North East	4	545,000	965	242
North West	1	231,900	452	452
Ontario (total)	32	12,028,900	19,327	604

*HNHB* Hamilton Niagara Haldimand Brant, *LHIN* Local Health Integration Network, *N/A* Not Applicable, *SU* Stroke Unit, *TIA* Transient Ischemic Attack

^a^Data source: Ontario Stroke Evaluation Report 2014 [17]

### Structure and organization

The majority of SUs were located on general medicine wards (*n* = 18; 56%), followed by neurology or rehabilitation wards (*n* = 9; 28%). Few SUs defined themselves as a dedicated stand-alone SU with their physical location not being associated with a specific specialty ward (*n* = 5; 16%). Half of the ASUs had no beds exclusively allocated for stroke patients; but had priority beds. The median number of beds in ASUs and ISUs was 11 (range 4-37) and 19 (range 4-28), with an average of 80% and 91% being occupied on a typical day, respectively.

Although the reported average acute length of stay (LOS) did not vary significantly with the type of SU (ASU = 9.1 ± 4.2 days, ISU = 8.2 ± 2.9 days), the full LOS was significantly longer in ISUs, due to rehabilitation services (the rehabilitation LOS was 21.0 ± 10.9 days). One fifth of SUs noted that waiting times for diagnostic services often increases LOS. Table 2 summarizes the structure and organization of SUs.

**Table 2:** Structure and organisation of SUs

	Acute SUs (*n* = 21)	Integrated SUs (*n* = 10)	Rehabilitation SU (*n* = 1)	Total SUs (*n* = 32)
Operation time *(mean ± sd, years)*	6.9 ± 3.9	8.6 ± 5.2	3 ± 0	7.3 ± 4.4
* <5 years (n, %)*	7 (33)	2 (20)	1 (100)	10 (31)
* ≥5 years (n, %)*	14 (67)	8 (80)	0 (0)	22 (69)
Geographic location *(n, %)*				
*General medicine*	14 (67)	4 (40)	0 (0)	18 (56)
*Neurology*	4 (19)	0 (0)	0 (0)	4 (12)
*Rehabilitation*	0 (0)	4 (40)	1 (100)	5 (16)
*Dedicated unit*	3 (14)	2 (20)	0 (0)	5 (16)
SUs with dedicated^a^ beds *(n, %)*	10 (48)	10 (100)	1 (100)	21 (66)
Number of beds *(median, range)*	11 (4-37)	19 (4-28)	6 (-)	13 (4-37)
Mean bed occupancy percentage on a usual day *(range)*	80% (50-100)	91% (60-100)	83% (-)	84% (50-100)
Acute LOS *(mean ± sd, days)*	9.1 ± 4.2	8.2 ± 2.9	-	8.8 ± 3.7
Rehabilitation LOS *(mean ± sd, days)*	-	21.0 ± 10.9	38 ± 0	23.6 ± 10.7
Responsible physician for patient care				
*Stroke Neurologist (n, %)*	6 (29)	1 (10)	0 (0)	7 (22)
*General neurologist (n, %)*	7 (33)	1 (10)	0 (0)	8 (25)
*Internist (n, %)*	13 (62)	8 (80)	0 (0)	21 (66)
*Family physician (n, %)*	3 (14)	5 (50)	1 (100)	9 (28)

Data was calculated after excluding missing values

*LOS* Length of Stay, *SUs* Stroke Units, *SD* Standard Deviation

^a^Beds that are exclusively allocated for stroke patients

### SUs staff composition, workload and specialized training

The majority of ASUs (*n* = 13, 62%) and ISUs (*n* = 8, 80%) indicated that an internist was the most responsible physician for patient care (Table 2). One third of the SUs lacked a general neurologist or stroke neurologists on staff (ASU: *n* = 6/21, ISU: *n* = 4/10). A core multidisciplinary team (nursing, physiotherapist (PT), occupational therapist (OT), speech language pathologist (SLP), social worker (SW) and clinical nutritionists) was available in all SUs, either as dedicated staff or for consultation. There was no difference between ASUs and ISUs in dedicated allied health personnel median full time equivalent (FTE) (0.5-0.8 FTEs per 10 beds). However, ASUs had substantially higher FTEs for nurses, educators and care managers (Table 3). Data regarding FTEs for non-dedicated staff was not collected.

**Table 3:** Composition and staffing details for multidisciplinary team in ASUs and ISUs

	Dedicated n (%)	Consultation n (%)	FTE per 10 bed^a^ median (range)	Patient to staff ratio
Acute SUs (*n* = 21)				
PT	10 (47.6)	11 (52.4)	0.8 (0.6-2.7)	12:1
OT	10 (47.6)	11 (52.4)	0.8 (0.5-2.0)	12:1
SLP	6 (28.6)	15 (71.4)	0.6 (0.3-1.8)	12:1
SW	7 (33.3)	14 (66.7)	0.5 (0.3-1.0)	-
Nutritionist	5 (23.8)	16 (76.1)	0.2 (0.2-1.0)	-
Pharmacist	5 (23.8)	16 (76.1)	1.0 (1.0-2.0)	-
Psychologist	-	7 (33.3)	-	-
RN	6 (28.6)	15 (71.4)	11.3 (1.0-17.0)	
*Day-shift*				4:1
*Night-shift*				6:1
RPN	4 (19.1)	13 (61.9)	11.0 (2.9-12.5)	
*Day-shift*				5:1
*Night-shift*				7:1
APN	5 (23.8)	3 (14.3)	0.8 (0.3-1.0)	-
Educator	6 (28.6)	15 (71.4)	0.4 (0.2-1.7)	-
Admin. staff	5 (23.8)	11 (52.4)	0.6 (0.1-1.3)	-
Care manager	6 (28.6)	12 (57.1)	1.0 (0.4-2.5)	-
Integrated SUs (*n* = 10)				
PT	6 (66.7)	3 (33.3)	0.8 (0.5-1.0)	12:1
OT	6 (66.7)	3 (33.3)	0.8 (0.5-1.0)	13:1
SLP	5 (55.6)	4 (44.4)	0.5 (0.3-0.6)	14:1
SW	5 (55.6)	4 (44.4)	0.5 (0.2-4.0)	-
Nutritionist	4 (44.4)	5 (55.6)	0.5 (0.2-0.6)	-
Pharmacist	3 (33.3)	6 (66.7)	0.8 (0.5-1.0)	-
Psychologist	-	3 (33.3)	-	-
RN	8 (88.9)	1 (11.1)	8.0 (3.5-13.0)	
*Day-shift*				4:1
*Night-shift*				7:1
RPN	7 (77.8)	1 (11.1)	4.2 (1.9-15.0)	
*Day-shift*				5:1
*Night-shift*				7:1
APN	2 (22.2)	3 (33.3)	0.3 (0.2-0.4)	-
Educator	2 (22.2)	7 (77.8)	0.9 (0.2-1.7)	-
Admin. staff	7 (77.8)	1 (11.1)	1.0 (0.4-1.8)	-
Care manager	6 (66.6)	3 (33.3)	0.4 (0.2-0.8)	-

Data was calculated after excluding missing values

*APN* Advanced Practice Nurse, *FTE* Full Time Equivalent, *OT* Occupational Therapist, *PT* Physiotherapist, *RN* Registered Nurse, *RPN* Registered Practical Nurse, *SLP* Speech Language Pathologist, *SW* Social Worker, *SUs* Stroke Units

^a^FTE presented for dedicated personnel only

Both acute and integrated SUs had nurse-to-patient ratios of 1:4-5 during the day and 1:6-7 during the night with no difference between weekdays and weekends. There was little difference in the allied health staff-to-patient ratios; ASUs =1:12 for PT, OT and SLP and in ISU there was 1:12 for PT; 1:13 for OT and 1:14 for SLP (Table 3).

Staff educational sessions that included both conferences and in-house talks in the SUs were attended on a monthly and yearly basis. One third of SUs (*n* = 10; 31%) indicated having budget allocated for training purposes, and only two thirds of the ISUs (*n* = 6) had nursing staff with specialized training in stroke care.

### SUs patient population and processes of care

**Fig. 2:**
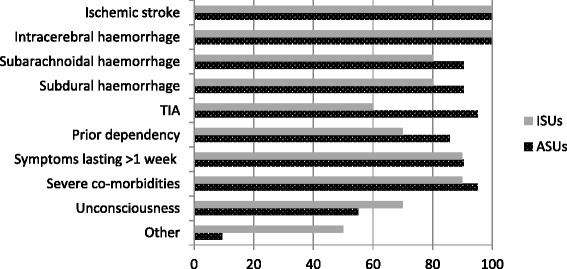
Patient population served in Stroke Units

Typically, patients were admitted with a clinical diagnosis of either ischemic or hemorrhagic stroke. ASUs were more likely to admit patients with transitory ischemic attack (TIA) compared with ISUs (*n* = 17, 80% vs *n* = 6, 60%). The majority of SUs (*n* = 18; 56.3%) did not admit unconscious patients (Fig. 2). Some SUs excluded patients with: symptoms lasting more than one week and severe co-morbidities, subdural hemorrhage and subarachnoid hemorrhage, patients with only transient symptoms, or prior dependency (Fig. 2).

Almost all SUs had access to computed tomography (CT) (*n* = 31, 97%), magnetic resonance imaging (MRI) (*n* = 30, 97%) and carotid Doppler ultrasound (*n* = 30, 97%). The access to on site surgical interventions was limited: neuro/-vascular surgery (*n* =12, 37.5%), interventional neurology (*n* = 13, 41%) and neuroradiology (*n* = 16, 50%). The administration of different valid assessment scales is summarized in Table 4. The vast majority of SUs had written protocols (*n* = 22; 69%) and standardized order sets (*n* = 32; 100%) in place to evaluate stroke-related impairments and further guide stroke care. Monitoring of skin surface areas for pressure ulcer prevention was reported in all SUs (Table 4).

**Table 4:** Processes of care in Stroke Units

*n (%)*	Acute SUs (*n* = 21)	Integrated SUs (*n* = 10)	Total (*n* = 32)^a^
Available diagnostic procedures			
*CT*	21 (100.0)	10 (100.0)	31 (96.9)
*MRI*	21 (100.0)	9 (90.0)	30 (93.8)
*Cerebral angiography*	18 (85.7)	8 (88.0)	26 (83.8)
*Carotid Doppler ultrasound*	20 (95.2)	10 (100.0)	30 (93.8)
Available on-site surgical therapy			
*Neuro/-vascular surgery*	9 (42.9)	3 (33.3)	12 (37.5)
*Interventional neurology*	10 (47.6)	3 (33.3)	13 (40.6)
*Interventional neuroradiology*	12 (57.1)	4 (44.4)	16 (50.0)
Standardized valid assessments scales			
*Barthel index*	8 (38.1)	6 (60.0)	14 (43.7)
*Canadian neurological scale*	18 (85.7)	7 (70.0)	25 (78.1)
*NIHSS*	14 (66.7)	7 (70.0)	21 (65.6)
*FIM*	20 (95.2)	10 (100.0)	30 (93.8)
*TORBSST*	10 (47.6)	5 (50.0)	15 (46.8)
*Braden scale*	20 (95.2)	10 (100.0)	31 (96.8)
Approaches to guide stroke care			
*Written protocols*	13 (65.0)	8 (80.0)	22 (70.9)
*Standardized order sets*	21 (100.0)	10 (100.0)	32 (100.0)
*Monitoring of pressure area*	21 (100.0)	10 (100.0)	32 (100.0)
Interdisciplinary team meeting ≥1/week	20 (95.2)	10 (100.0)	30 (93.8)
Goal setting	19 (90.5)	9 (90.0)	29 (90.6)
Creation of individualized care plans	12 (57.1)	10 (100)	23(71.8)
Timing of initial discharge assessment			
*< 24 h*	7 (33.3)	2 (20.0)	9 (28.3)
*1-2 d*	6 (28.6)	5 (50.0)	11 (34.4)
*3-4 d*	5 (23.8)	3(30.0)	8 (25.0)
*< 4 d*	3 (14.3)	0 (0.0)	3 (12.5)

*CT* Computed Tomography, *FIM* Functional Independence Measure, *MRI* Magnetic Resonance Imaging, *NIHSS* National Institute Health Stroke Scale, *TORBSST* Toronto Bedside Swallowing Screening Test, *SUs* Stroke Units

^a^Total SUs column includes information on the Rehabilitation unit (*n* = 1) as well

Early discharge assessment (within 48 h) was conducted in nearly two thirds of SUs (*n* = 20; 62%). Rehabilitation services were being provided on site in ISUs. Formal interdisciplinary team meetings were being conducted at minimum once a week in all but two SUs. During the meetings a goal setting (*n* = 29, 91%) and individualized rehabilitation care plans (*n* = 23, 72%) were discussed by majority of the units.

All SUs routinely provided educational material to patients and caregivers with information on stroke and rehabilitation, risk management, and availability of community resources. The most common formats of education were one-on-one teaching modules and pamphlets.

All core elements of SU care (geographically defined unit, multidisciplinary specialized staff, in-house diagnostic services, administration of valid assessment tools and implementation of standardized protocols, team meetings, rehabilitation and patient education) were addressed by 16 out of 32 units (50%) (Table 5). There was no noticeable clustering of deficient core elements in certain SUs, or in particular geographic regions (i.e. by LHIN). The main areas for improvement included specialized nurse training in stroke care (met by 26 units), provision of individualized rehabilitation care plans (met by 23 units), and early discharge assessment (met by 20 units) conducted within 48 h of admission.

**Table 5:** Summary of core elements met by SUs

*Core elements for SUs*	SU, n (%)
Geographically defined unit	32 (100.0)
Multidisciplinary team (physician, nurse, OT/PT/SLP/clinical nutritionist)	32 (100.0)
Advance training in stroke management^a^	26 (81.3)
Acute imaging (MRI/CT)	32 (100.0)
Valid scales (CNS/NIHSS/FIM)	30 (93.8)
Standardized order sets/protocols/algorithms	32 (100.0)
Interdisciplinary team meeting ≥1 per week	31 (96.9)
Creation of individualized rehabilitation care plans	23 (71.8)
Goal setting	29 (90.6)
Early discharge assessment (<48 h of admission)	20 (62.5)
Patient/carer education	32 (100.0)
In place rehabilitation (*for Integrated SUs only*)	10 (100.0)
All core elements met	16 (50.0)

Core elements were adopted from the Canadian Best Practices Recommendation

*CNS* Canadian Neurological Scale, *CT* Computed Tomography, *FIM* Functional Independence Measure, *MRI* Magnetic Resonance Imaging, *NIHSS* National Institute Health Stroke Scale, *OT* Occupational Therapist, *PT* Physiotherapist, *SLP* Speech Language Pathologist, *SUs* Stroke Units

^a^Data on nurses training only

## Discussion

We conducted a comprehensive survey of 32 SUs in Ontario, which included 21 ASUs, 10 ISUs and one RSU. According to the Canadian Best Practice Recommendations for Stroke Care, SUs should consist of a geographically defined unit with a dedicated multidisciplinary team with specialized expertise in stroke and standardized processes of care and resources [8]. The study revealed regional variation in access to SUs. We also found that many of the core components of SUs are in place, however half of the SUs in Ontario do not meet all criteria.

### SU regional distribution, structure and organization

This study demonstrated that the SUs distribution varied between regions, with no SU available in the Central West LHIN. There was a greater than six-fold difference in stroke/TIA population served by the SUs, ranging from 242 in the North East LHIN to 1480 in the Mississauga Halton LHIN. Potential disparity in access to SU care might be supported by the fact that only 19% (27 out of 142) of acute care hospitals in Ontario had a SU in 2010-2011, and only 38.3% of admitted stroke patients were treated in SUs [18]. The access was dramatically lower compared with European countries, (2010-2011) where over 60% of stroke patients received care in SUs [19, 20]. Evidence from clinical trials and observational studies suggests that stroke care provided in geographically defined SUs is associated with a 20% lower chance of death relative to care in general wards [4, 6]. In the current study, all 32 identified SUs had a geographically defined location, and the majority were based in a general medicine ward with a median of 13 beds. At the time of the survey, Canadian recommendations did not require SUs to be exclusive to stroke patients, nor have all of the resources in place; however, recommendations did require SUs to be in one geographic location [17]. Later in 2014, the OSN revised the definition of SUs, which included additional criteria related to bed allocation and staff. According to the new definition “a stroke unit is a geographical unit with identifiable co-located beds that are occupied by stroke patients 75% of the time and have a dedicated interprofessional team with expertise in stroke care including, at a minimum, nursing, physiotherapy, occupational therapy and speech-language pathology” [17]. Though our questionnaire did not specifically ask for the number of beds occupied exclusively by stroke patients, we can speculate that the ASUs with no dedicated beds for stroke patients (half of ASUs) might fail to meet that criterion. This could partially explain decreased number of SUs (*n* = 14) identified in 2014 by OSN that satisfied the new definition [17]. European guidelines are more restrictive and require SUs to be exclusive for stroke and TIA patients only, and not to admit patients with other disorders [21]. The goal is to train staff to better address the needs of stroke patients and gain specialized skills and expertise, which could translate into improved patient outcomes [21]. In the observational study looking into mortality for 26 000 stroke patients, Saposnik et al., concluded that seven day mortality was 9.5% for hospitals with a volume of less than 50 stroke patients per year, 7.3% for a volume of 100-199 per year, and 6.0% for a volume of more than 200 (<0.001) [22].

### SUs staff composition, workload and specialized training

Nursing and allied health staff was available in all units, either as dedicated staff or on a consultation basis. However, there still might be room for improvement, such as adjustment in staffing levels and an optimization in the staff-to-patient ratio. FTE for allied health professionals (~0.5-0.8 FTE per type per 10 beds) was somewhat lower than reported in European studies (PT/OT 1–2, for SLP 0.2-0.6 [23, 24]). This is also supported by our finding of the high patient-to-therapist ratio (12-14:1), which was almost double than reported in US clinics (5-8 :1) [25]. Nursing allocation (8-10 FTE per 10 beds) was comparable to other studies. Across 92 surveyed SUs in England, a median of 11 nurses per 10 beds was recorded [23]. Recommendations regarding nurse staffing levels vary across countries and depend on local contexts and delivery models. Scottish Guidelines recommend having 10 nurses [24], while European Stroke Organization requires minimum of 15 nurses per 10 monitored beds [21]. Using data from Canadian practice and several other studies, Phillips et al., derived the following FTE allocation (per 10 beds) for multidisciplinary staff: RN –14; OT/PT/SLP – 1.0 each; and SW – 0.5 [26]. In our study only a few SUs reported on availability of an advanced practice nurse (APN) (*n* = 5, 16%), which according to the Brain Attack Coalition is a vital member of a stroke team who provides support with patient care, educational programs, research activities and quality assurance [27].

Stroke-focused training for nurses might be considered as another major area of improvement, since only two thirds of the ISUs included nurses who received specialized training in stroke care. International stroke experts consider nurses trained in stroke management as an absolutely integral element of care [19]. The German Stroke Society has established a certified stroke nursing curriculum with over 200 h of training [19]. Lack of specialized nurse training in Ontario might be explained by scarce resources, since only one third of ISUs had specific funds allocated for training purposes. Continuing stroke education is important not only for nurses, but also for other medical staff, since internists were the most frequently reported responsible physicians for patient care. Our questionnaire did not capture information on physician training in stroke management; however general neurologists or stroke neurologists were more likely to receive such training, rather than internists. In Ontario, it was found that one third of the SUs lacked a general neurologist or stroke neurologists on staff. In contrast, 196 out of 200 SUs in Germany were run by neurologists and only four by an internist [19]. In a survey conducted by the US Brain Attack Coalition, leaders and experts in cerebrovascular disease rated having a stroke neurologist on staff as an important element of a stroke centre [27]. Furthermore, in a nationwide prospective observational study, Goldstein et al. revealed that patients with ischemic stroke who were treated by neurologists experienced improved outcomes, such as less dependency or mortality (OR = 0.64; 95% CI 0.45 to 0.92) than patients who were cared for by non-neurologists [28].

### Patient population and processes of care

In addition to ischemic and haemorrhagic stroke patients, most of the acute and integrated SUs also admitted patients with transient symptoms, prior dependency, and severe co-morbidities. Recommendations indicate that all patients with a suspected diagnosis of stroke urgently undergo brain imaging (CTs or MRIs) [8, 21, 27]. Although specific diagnostic imaging necessary for stroke patient management was highly available in SUs, one fifth of SUs mentioned that waiting times for diagnostic services often increase LOS. Although our study questionnaire did not specifically address acute imaging wait times,, only 22% of stroke patients received a scan within one hour of arrival according to the Canadian Stroke Network 2011 report [10]. This emphasizes the need for written protocols and standardized order sets that will prioritize the access of stroke patient to diagnostic services and time-sensitive treatments. Phillips et al. acknowledged that having standardized order sets in their SU in Halifax not only improved recording of the information and its accessibility to the stroke team, but also facilitated interdisciplinary assessment and treatment [29]. Although all SUs mentioned the availability of standardized order sets, only two thirds of SUs had written protocols in place to guide stroke care.

It was encouraging to report the administration of Braden Scale for pressure ulcer monitoring as a part of daily routine in all SUs. This may play an important role in pressure ulcer prevention [30], as pressure ulcers increase health services utilization and impair quality of life [31]. The administration of validated scales to monitor mental and motor functions of stroke patients (Canadian Neurological Scale, the National Institutes of Health Stroke Scale and Functional Independence Measure [32–34]) was high in SUs (66%–100%), and was comparable to other surveys conducted by Langhorne et al. [15].

Early discharge services and rehabilitation are cornerstones of stroke patient management. In the meta-analysis of 11 trials from six countries (Australia, Canada, Norway, Sweden, Thailand, UK) Langhorne et al., showed that early supported discharge reduced the risk of death or long-term dependency (OR 0.79 95% CI 0.64 to 0.97) in stroke patients, as well as shortened LOS [35]. Meanwhile, Kwakkel et al. reported positive association between intensity of rehabilitation therapies and functional recovery following stroke [36]. Though our questionnaire was not designed to capture patient level data, it seems that rehabilitation services need to be improved, since not all units conducted early discharge assessment (*n* = 20, 63%) or provided individualized rehabilitation care plans (*n* = 23, 72%).

A Cochrane review by Smith et al. assessed the effectiveness of strategies for information provision to stroke patients and their caregivers. They supported routine provision of information; however, there was no preferred method of communication [37]. In this study, SU managers mentioned that the most common format of patient education were pamphlets and one-to-one teaching modules.

Based on our survey results the following areas should be prioritized to improve stroke care in Ontario: i) optimization of staffing level and intensity for nurses and allied health professionals, ii) addressing the lack of general neurologists and stroke neurologists in SUs iii) specialized stroke care training for a multidisciplinary team, iv) priority bed allocation for stroke patients, v) provision of individualized rehabilitation plans and early discharge assessment.

### Strength and limitations

Employing two sampling strategies for this pan-provincial scan, we were able to identify two additional SUs in addition to those listed by the OSN in 2011 [13]. With our 100% response rate we were able capture core characteristics, organisational structures and care processes of the 32 identified SUs. The first limitation of the study was that it was based on feedback from SU managers and consequently, interpretations of staff-to-patient ratios should be made with caution. In our study, dedicated staffing levels and full-time equivalent (FTE) information for stroke teams were difficult to obtain, since different levels of cross-cover with other non-stroke services (e.g. general ward, neurology) were reported. In addition, staffing ratios may not be easily comparable to other jurisdictions, since studies defined and calculated staffing levels in various ways.

Secondly, there was potential underreporting of some procedures; i.e. managers of SUs might had been unaware of the administration of specific diagnostic tools for an impairment assessment). The survey questionnaire did not cover all important components of stroke care, i.e. assessment of treatment package, early mobilisation practices, and complication management.

Finally, as noted above, the survey was conducted before the OSN revised the definition of a SU in 2014, which introduced new criteria on co-allocated beds and its occupancy rate by stroke patients [17]. This led to a reduction in the number of available SUs in Ontario to 14, which all satisfied the new criterion.

## Conclusion

This study illustrated the heterogeneous structure of SUs in Ontario and signalled potential disparity in access to SU care. Only half of the identified SUs met all core criteria set forth by the Canadian Stroke Strategy Guide to the Implementation of Stroke Unit Care 2009 [9]. Finally, this study has revealed important areas for improvement in SU care, such as stroke care training for a multidisciplinary team, adjustment in staffing level and intensity for nurses and allied health professionals, bed allocation for stroke patients, and the provisioning of individualized rehabilitation plans and early discharge assessment.

## Additional files

Additional file 1: Organized Inpatient (Stroke Unit) Care Questionnaire. Blank version of the survey questionnaire. (DOCX 24 kb)
Additional file 2: Distribution of identified Stroke Units across Local Health Integrational Networks. This file lists all identified and surveyed stroke units across Local Health Integrational Networks. (DOCX 16 kb)

## Abbreviations

ASU: Acute stroke unit
CI: Confidence interval
CT: Computed tomography
FTE: Full time equivalent
ISU: Integrated stroke unit
LHIN: Local health integrational network
LOS: Length of stay
MRI: Magnetic resonance imaging
OR: Odds ratio
OSN: Ontario stroke network
OT: Occupational therapist
PT: Physiotherapist
RSU: Rehabilitation stroke unit
SLP: Speech language pathologist
SU: Stroke unit
SW: Social worker
TIA: Transitory ischemic attack
UK: United Kingdom
US: United States
