# Supplementary material for: The characteristics of stroke units in Ontario: a pan-provincial survey
Source: BMC Health Serv Res. 2017 Feb 21;17:154. doi: 10.1186/s12913-017-2099-1 (PMC5320701; doi:10.1186/s12913-017-2099-1)
Supplement: Additional file 1: — Organized Inpatient (Stroke Unit) Care Questionnaire. Blank version of the survey questionnaire. (DOCX 24 kb) [file 12913_2017_2099_MOESM1_ESM.docx]

**Organized Inpatient (Stroke Unit) Care Questionnaire:**

1. **General information:**
   1. Name of organization (print full name, overarching institution, and site/campus/department):
   2. Year stroke unit opened (yyyy):
   3. *What is the physical location of the stroke unit?* (circle one):
      1. Hospital (circle appropriate ward[s]):
         1. Geriatric medicine
         2. Neurology
         3. General medicine
         4. Other (print): _____
      2. Dedicated stroke unit not associated with a specific specialty or sub-specialty ward
   4. Date questionnaire was administered (dd/mm/yyyy):
   5. Interviewer name (first name, last name): _____
   6. Stroke unit contact (interviewee) information (role/title only): _____

­­

1. **Type of stroke unit (circle one):**
   1. *Please indicate which type of stroke unit most closely applies to your stroke unit from the following five types that I will describe to you now:*
      1. **Acute stroke unit:** A specialized, geographically defined hospital unit dedicated to the management of stroke patients during the first 7 to 10 days, or longer, following an acute stroke event and staffed by an interprofessional team.
      2. **Integrated stroke unit:** A specialized, geographically defined hospital unit dedicated to the management of stroke patients. The unit provides both acute and rehabilitation care to patients during their inpatient stays following a stroke.
      3. **Stroke rehabilitation unit:** A specialized, geographically defined rehabilitation unit dedicated to the rehabilitation of stroke patients.
      4. **Mixed rehabilitation ward:** A multidisciplinary team in a ward including specialist nursing staff that provides a generic rehabilitation service, but not exclusively caring for stroke patients.
      5. **Mobile stroke team:** A multidisciplinary team excluding specialist nursing staff that provides care in a variety of settings.
   2. *Are stroke patients cared for in wards other than the stroke unit or ICU? (yes/no)*
      1. How many (non-ICU) beds are typically looked after outside of the stroke unit? Number:_____
2. **Admission to stroke unit**
   1. *What is the stroke unit’s current practice on patient admission to the stroke unit?* *Please rank the following three options in order of most to least common.* (*Note to interviewer: 1 = most common, 3 = least common*):
      1. Directly from the emergency room: _____
      2. Transfer from another ward: _____
      3. Transfer from the emergency room to a ward, then from a ward to the stroke unit: _____
   2. Time spent in the stroke unit:
      1. *Are patients cared for exclusively by the stroke unit for the full duration of their acute length of stay? (yes/no)*
         1. *If no*, *do patients spend at least 75% of their acute length of stay in the stroke unit? (yes/no)*
      2. *What unit (ward) are patients transferred to after they have been discharged from the stroke unit?* (print): ­­­­­­­­­­­­­­_____
   3. Information on patients admitted to the stroke unit:
      1. *What is the full duration for the average length of stay in the stroke unit in your opinion?* (days): _____
         1. *What is the average length of stay in the stroke unit for acute care?* (days): _____
         2. *If applicable, what is the average length of stay in the stroke unit for rehabilitation care (or rehabilitation LOS = full duration LOS – acute LOS)?* (days): _____
      2. *What is the stroke unit’s policy on the types of patients that can be admitted to the stroke unit? Please indicate yes or no to the following type of stroke patients (Note to interviewer: use space below if a description is required):*
         1. *Types of stroke:*
            1. Ischaemic stroke (yes/no)
            2. Primary intracerebral haemorrhage (yes/no)
            3. Subarachnoid haemorrhage (yes/no)
            4. Subdural haemorrhage (yes/no)
            5. Transient stroke symptoms (yes/no)
         2. Other Conditions:
            1. Prior dependency (patients requiring help for (Instrumental) Activities of Daily Living (ADLs, or IADLs) (yes/no)
            2. Symptoms lasting more than one week (yes/no)
            3. Severe co-morbidity (yes/no)
            4. Unconsciousness (yes/no)
         3. Other (print): _____
3. **Resource Characteristics**
   1. Bed occupancy
      1. Do you have dedicated beds? (yes/no)
         1. *If ‘yes’, what is the maximum number of beds allocated to the stroke unit?* Number: _____
         2. *If ‘yes’, on a usual day, how many of these allocated beds are full?* Number: _____
   2. *What type of physician is most responsible for patients in the stroke unit out of the following list of staff physicians? (You may circle more than one).*
      1. Stroke neurologist
      2. General neurologist
      3. Internal medicine
      4. Family physician
      5. Geriatrician
      6. Physiatrist
      7. Other (print): _____

- 1. *How many hours per week is the most responsible physician physically present in the stroke unit? Number:* _____
  2. Which physicians staff the stroke unit during the day? What percentage of the time do physicians sees patients on the unit? (Percentages should add up to 100%)
     1. Stroke neurologist (yes/no) ______%
     2. General neurologist (yes/no) ______%
     3. Internal medicine (yes/no) ______%
     4. Family physician (yes/no) ______%
     5. Geriatrician (yes/no) ______%
     6. Physiatrist (yes/no) ______%
     7. Other: _______________________ ______%
  3. Which physicians staff the stroke unit during after-hours (night and weekend care)? What percentages of the time do physicians see patients on the unit? (Percentages should add up to 100%)
     1. Stroke neurologist (yes/no) ______%
     2. General neurologist (yes/no) ______%
     3. Internal medicine (yes/no) ______%
     4. Family physician (yes/no) ______%
     5. Geriatrician (yes/no) ______%
     6. Physiatrist (yes/no) ______%
     7. Other: _______________________ ______%
  4. *What is the current staffing complement? Please indicate all that apply from the following list and indicate the proportion of patients that could see the following clinical staff if they needed to*:
     1. Specialist(s)
        1. Stroke neurologist __ FTE (dedicated/consultation/N/A)
        2. General neurologist __ FTE (dedicated/consultation/N/A)
        3. Internal medicine __ FTE (dedicated/consultation/N/A)
        4. Family physician __ FTE (dedicated/consultation/N/A)
        5. Geriatrician __ FTE (dedicated/consultation/N/A)
        6. Physiatrist __ FTE (dedicated/consultation/N/A)
        7. List type_____ __ FTE (dedicated/consultation/N/A)
     2. (Neuro)psychologist(s) __ FTE (dedicated/consultation/N/A)
     3. Pharmacist(s) __ FTE (dedicated/consultation/N/A)
     4. Dietician(s) __ FTE (dedicated/consultation/N/A)
     5. Physiotherapist(s) __ FTE (dedicated/consultation/N/A)
     6. Occupational therapist(s) __ FTE (dedicated/consultation/N/A)
     7. SLP(s) __ FTE (dedicated/consultation/N/A)
     8. Recreation therapist(s) __ FTE (dedicated/consultation/N/A)
     9. Advanced practice nurse(s) __ FTE (dedicated/consultation/N/A)
     10. Registered practical nurse(s) __ FTE (dedicated/consultation/N/A)
     11. Registered nurse(s) __ FTE (dedicated/consultation/N/A)
     12. Social worker(s) __ FTE (dedicated/consultation/N/A)
     13. Educator(s) __ FTE (dedicated/consultation/N/A)
     14. Administrative Assistant(s) __ FTE (dedicated/consultation/N/A)
     15. Care manager __ FTE (dedicated/consultation/N/A)
     16. Other (print): ______________ __ FTE (dedicated/consultation/N/A)
     17. Other (print): ______________ __ FTE (dedicated/consultation/N/A)
     18. Other (print): ______________ __ FTE (dedicated/consultation/N/A)
  5. What is the typical therapists-to-patients ratio?
     1. Physiotherapist(s):
        1. Day: ________________________
        2. Night: ______________________
        3. Weekends: ___________________
     2. Occupational therapist(s):
        1. Day: ________________________
        2. Night: ______________________
        3. Weekends: ___________________
     3. Speech and language pathologist(s):
        1. Day: ________________________
        2. Night: ______________________
        3. Weekends: ___________________
  6. Nursing model:
     1. What type of nurses are regularly present in the stroke unit?
        1. Nurse practitioner/Advanced practice nurse (yes/no)
        2. Registered practical nurse (yes/no)
        3. Registered nurse (yes/no)
     2. What is the typical nurse-to-patient ratio?
        1. Nurse practitioner/Advanced practice nurse
           1. Day: ________________________
           2. Night: _______________________
           3. Weekends: ___________________
        2. Registered practical nurse
           1. Day: ________________________
           2. Night: _______________________
           3. Weekends: ___________________
        3. Registered nurse
           1. Day: ________________________
           2. Night: _______________________
           3. Weekends: ___________________
     3. Have all of these nurses received specialized training in the care of stroke patients? (yes/no)
  7. *Which of the following diagnostic equipment do stroke unit patients have access to?*
     1. Electrocardiogram (ECG) (yes/no)
     2. CT (computed tomography) scanner (yes/no)
     3. Plain film radiography (x-ray) (yes/no)
     4. MRI/MRA unit (yes/no)
     5. Cerebral angiography using contrast (yes/no)
     6. Echocardiogram (yes/no)
     7. Holter monitor (yes/no)
     8. Carotid doppler ultrasound (yes/no)
     9. Electroencephalogram (EEG) (yes/no)
     10. Other (print) _____

- 1. *How often does wait time for the above diagnostic services increase patients’ length of stay in the hospital? (circle one):*
     1. Never
     2. Occasionally
     3. Often
  2. *Is on-site neurological surgery available to patients?* (yes/no)
  3. *Is on-*site *neurovascular surgery available to patients? (yes/no)*
  4. *Do patients have access to interventional neurology?* (yes/no)
  5. *Do patients have access to interventional neuroradiology? (yes/no)*
  6. *What are the interprofessional team meetings or rounds held (specify formal and informal):*

Weekly:______________________________________________________

Monthly:______________________________________________________ Yearly:_______________________________________________________

- - 1. *What is the number of formal interprofessional team meetings or rounds per week?*: Number: _____
       1. *Are these formal interprofessional team meetings attended by:*
          1. *Medical staff? (yes/no)*
          2. *Patients? (yes/no)*
          3. *Caregivers?* (yes/no)
       2. *Do the formal interprofessional team meetings include goal setting?* (yes/no)
       3. *Do formal interprofessional team meetings include creating individualized, comprehensive rehabilitation plans for patients?* (yes/no)
       4. *Which staff other than nurse and medical staff routinely attend the formal interprofessional team meetings or rounds?* (list): _____
    2. *What is the number of informal meetings or rounds held each week?*

Number:_____

- - - 1. *Are these informal meetings attended by:*
         1. *Patients? (yes/no)*
         2. *Caregivers?* (yes/no)
  1. *Education is routinely provided to patients and carers in which of the following areas?*
     1. Information on stroke (yes/no)
     2. Information on rehabilitation and recovery (yes/no)
     3. Available community resources (yes/no)
     4. Information on medications (yes/no)
     5. Stroke support groups (yes/no)
     6. Risk factor modifications (yes/no)
     7. Smoking cessation (yes/no)
  2. *Information to patients and carers is routinely provided in which of the following formats*:
     1. Pamphlets (yes/no)
     2. Videos (yes/no)
     3. One-to-one teaching (yes/no)
     4. Web or e-learning (yes/no)
     5. Other (print): ­­­­­­­­­­­­­_____
  3. Staff education:
     1. What type of staff education is provided or attended (i.e. teaching sessions, academic or regional stroke education programs):

Weekly:________________________________________________

Monthly:________________________________________________ Yearly:_________________________________________________ *How many teaching sessions were organized or supported by the stroke unit in the past year?* ­­­Number:_______________________

- - - - 1. *Please list the topics that were covered by these teaching sessions* (indicate Not Applicable if there were no teachings in the past year): _____
      1. *Does the stroke unit have a budget for staff to attend additional training sessions?* ­­­­(yes/no)
      2. *How many staff in the stroke unit have attended academic or regional stroke education programs in the past year?*

Number:_____

- - - 1. *How many opportunities were available to staff to attend academic or regional stroke education programs?*

Number:_____

How many stroke education programs per year in house? Number:_____

- 1. Performance improvement initiatives:
     1. *Does your facility undertake process or performance improvement initiatives (i.e. quality improvement framework)? (yes/no)*
        1. If ‘yes’, how do they undertake quality improvement in the stroke unit?
           1. Auditing (i.e. a review of process and performance indicators) (yes/no) Frequency:______________________
           2. Monitoring (i.e. a review of performance indicators only) (yes/no) Frequency:_______________________________
     2. Does your facility perform surveys of patient satisfaction specifically for stroke patients? (yes/no)
     3. Does your facility receive feedback from stroke support groups? (yes/no)

1. **Processes of Care**
   1. Nursing and therapy assessments include:
      1. Monitoring of pressure areas (including pressure risk scores) (yes/no)
      2. Use of standardized, valid assessment scales (tools) to evaluate stroke-related impairments and functional status:
         1. Barthel Index (yes/no)
         2. Canadian Neurological Score (CNS) (yes/no)
         3. National Institutes of Health Stroke Scale (NIHSS) (yes/no)
         4. Alpha Functional Independence Measure (FIM) (yes/no)
         5. Orpington Stroke Scale (yes/no)
         6. Toronto Bedside Swallowing Screening Test (TORBSST) (yes/no)
         7. Braden Scale (yes/no)
         8. Other (print): _____
      3. Neurological monitoring (i.e. after the initial use of the above assessment scales, are any of these scales or other scales used again for monitoring) (yes/no)
   2. *Which of the following interventions are normally used in the stroke unit?*:
      1. Written rehabilitation protocols to guide acute stroke care/rehabilitation (yes/no)
      2. Care pathways (algorithms) to guide acute stroke care/rehabilitation (yes/no)
         1. *If ‘yes’, please list the care pathways (algorithms) used (e.g., deep vein thrombosis [DVT] prophylaxis, swallowing).*

_________________________________________________

- - 1. Standardized order sets in place to guide acute stroke care/rehabilitation (yes/no)

1. **Discharge planning and follow-up**
   1. *Is a pre-discharge assessment of patients’ homes conducted as part of an early discharge program?* (yes/no)
      1. *If ‘yes’, what percent (proportion) of patients receive this service?*

*_____*

- 1. *What is the usual number of days between the date-of-admission to the time-to-assessment for discharge needs (days)?* (circle one):
     1. *Within 24 hours*
     2. *1-2 days*
     3. *3-4 days*
     4. *4 or more days*
  2. *When patients are discharged for inpatient rehabilitation services, are patients transferred to a new bed?* (yes/no)
     - 1. *If ‘yes’, which ward is the patient discharged to before inpatient rehabilitation services begin?* (print): ­­­­­­­­­­­­_____
