# Supplementary material for: The characteristics of stroke units in Ontario: a pan-provincial survey
Source: BMC Health Serv Res. 2017 Feb 21;17:154. doi: 10.1186/s12913-017-2099-1 (PMC5320701; doi:10.1186/s12913-017-2099-1)
Supplement: Additional file 2: — Distribution of identified Stroke Units across Local Health Integrational Networks. This file lists all identified and surveyed stroke units across Local Health Integrational Networks. (DOCX 16 kb) [file 12913_2017_2099_MOESM2_ESM.docx]

**Distribution of identified Stroke Units across Local Health Integrational Networks**

| **LHIN** | **Clinic name** |
| --- | --- |
| **Erie St. Clair** | 1. Chatham Kent Health Alliance 2. Bluewater Health - Sarnia General Site 3. Hotel-Dieu Grace Hospital |
| **South West** | 1. London Health Sciences Centre |
| **HNHB** | 1. Hamilton Health Sciences Corporation 2. Niagara Health System |
| **Waterloo Wellington** | 1. Grand River Hospital Corporation |
| **Mississauga Halton** | 1. Trillium Health Centre |
| **Central West** | N/A |
| **Central** | 1. Humber River Regional Hospital - Humber Memorial (Church site) 2. Humber River Regional Hospital - York-Finch 3. North York General Hospital 4. Southlake Regional Health Centre |
| **Central East** | 1. The Scarborough Hospital 2. Mackenzie Health (formerly York Central Hospital) 3. Peterborough Regional Health Centre 4. Lakeridge Health Corporation |
| **Toronto Central** | 1. St. Michael's Hospital 2. Toronto Western hospital 3. Toronto East General Hospital 4. Sunnybrook & Women's College Health Sciences Centre |
| **North Simcoe Muskoka** | 1. Royal Victoria Hospital of Barrie |
| **South East** | 1. Kingston General Hospital 2. Quinte Healthcare Corporation |
| **Champlain** | 1. Pembroke Regional Hospital Inc. 2. Glengarry Memorial Hospital 3. The Ottawa Hospital - Civic Site 4. The Ottawa Hospital - General Site |
| **North East** | 1. Sault Area Hospital - Sault Ste. Marie 2. North Bay Regional Health Centre 3. Timmins & District General Hospital 4. Health Sciences North - Ramsey Lake Health Centre |
| **North West** | 1. Thunder Bay Regional Health Sciences Centre |

HNHB - Hamilton Niagara Haldimand Brant
